# Pseudoverrucous Papules and Nodules in the Perianal Area: A Case Report

**DOI:** 10.7759/cureus.81945

**Published:** 2025-04-09

**Authors:** Ahmed Kurdi, Reeman Aljohani, Badr Aljohani, Taif Tharwat, Danah Alquwayzani, Raghad Alahmadi

**Affiliations:** 1 Department of Dermatology, King Salman bin Abdulaziz Medical City, Madinah, SAU; 2 Department of General Medicine, King Fahad Hospital, Madinah, SAU; 3 Department of Dermatology, Heraa General Hospital, Makkah, SAU; 4 College of Medicine, King Faisal University, Alahsa, SAU; 5 College of Medicine, Taibah University, Madinah, SAU

**Keywords:** irritant dermatitis, perianal lesion, pseudoverrucous papules and nodules, skin barrier, verrucous lesion

## Abstract

Pseudoverrucous papules and nodules (PPN) is a rare form of irritant contact dermatitis caused by chronic exposure to moisture and irritants. It commonly affects individuals with urostomy sites, perianal dermatitis, or peristomal irritation due to incontinence. Clinically, PPN presents as flat-topped, erythematous papules and verrucous nodules, which can resemble infectious or neoplastic conditions, making diagnosis challenging. This case report describes a 13-year-old boy with trisomy 21, imperforate anus, Hirschsprung’s disease, and chronic kidney disease who developed persistent perianal and peristomal lesions due to prolonged exposure to moisture. The patient was managed with frequent diaper changes, skin aeration, and topical treatments, including zinc oxide and tacrolimus 0.03%, leading to gradual resolution over four months. This case highlights the importance of recognizing PPN and implementing appropriate preventive and therapeutic measures to ensure optimal patient care.

## Introduction

Pseudoverrucous papules and nodules (PPN) is a rare condition considered a form of irritant contact dermatitis. The irritation leads to the formation of papules and nodules, which can present with distinct clinical features [1]. It is characterized by round, flat-topped, hyperpigmented erythematous papules and nodules [2,3]. Initial reports of this condition linked it primarily with urostomy sites. However, it may also appear in other areas, particularly the perianal area, secondary to urinary incontinence or encopresis [4]. PPN can closely mimic various infectious, inflammatory, and neoplastic disorders [1]. Multiple factors, such as irritant type, exposure duration, and environmental conditions, influence the severity of dermatitis in PPN. These factors act particularly in an overwarmed and macerated environment [5].

There are limited case reports of PPN on the perianal skin or in the peristomal region surrounding colostomies and urostomies. The rarity of these lesions is coupled with diagnostic challenges, which may lead to misdiagnosis [3]. Dermal protective agents alone are insufficient for treatment, as lesion regression occurs solely after the elimination of contributing irritants [1]. Below, we present a case of a 13-year-old who developed this rare form of PPN.

## Case presentation

**Figure 1:**
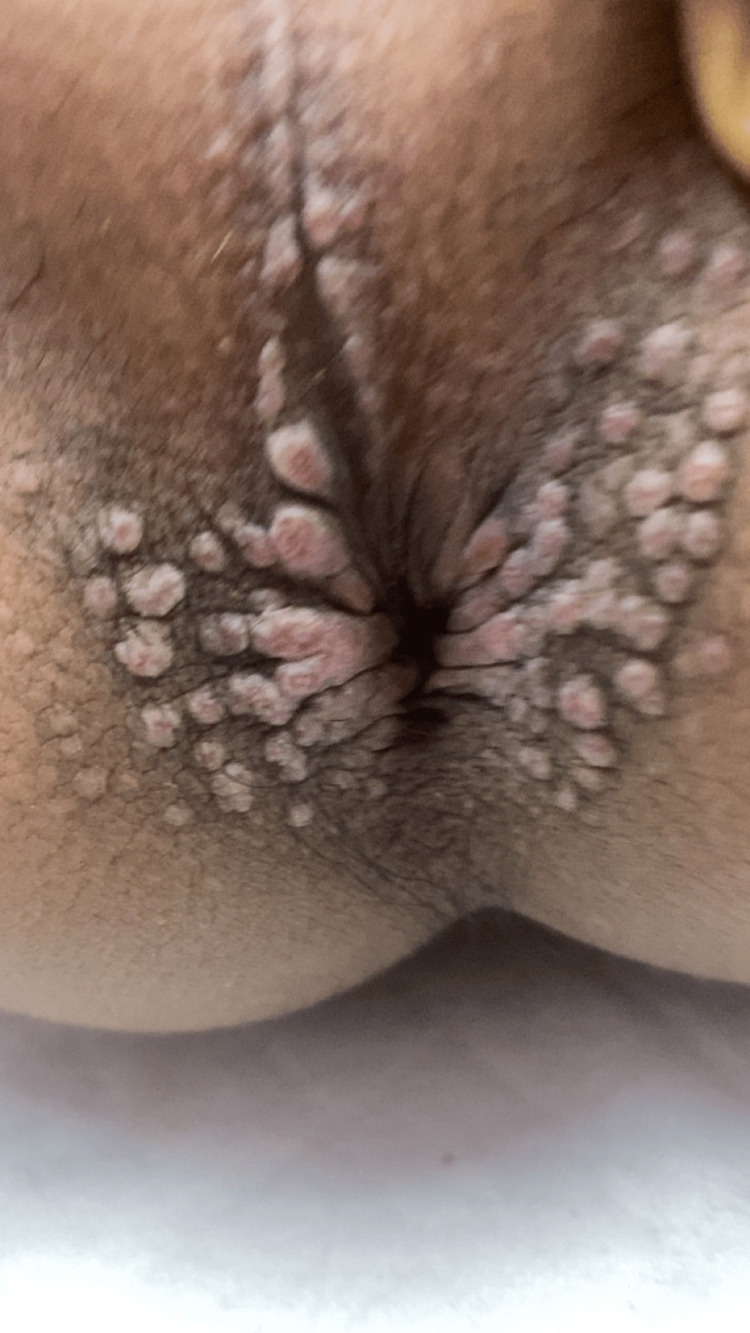
Pseudoverrucous papules and nodules (PPN). Multiple flat-topped and round, shiny, erythematous, and moist papules ranging in size from 4 to 15 mm, along with a few verrucous nodules. Several lesions had coalesced to form plaques extending to the posterior part of the scrotum.

A 13-year-old boy presented to the dermatology clinic with a chief complaint of a persistent skin lesion around the anal area for the past four months. He is a known case of trisomy 21 and has a history of multiple congenital anomalies and comorbidities. The patient has been incontinent of stool since birth and has required the continuous use of diapers. At birth, he was diagnosed with an imperforate anus, which was surgically managed with an anoplasty performed by the pediatric surgery team. Additionally, he was diagnosed with Hirschsprung’s disease and subsequently underwent a pull-through procedure. He also has a neurogenic bladder and bilateral severe hydroureteronephrosis, for which he is currently on hemodialysis due to chronic kidney disease (CKD). Medical history was free of any sexually transmitted diseases, trauma, surgery, radiotherapy, or infection. The patient had previously been treated with topical antifungals for similar skin lesions, with only mild improvement. The laboratory profile was non-reactive for human immunodeficiency virus (HIV), human papillomavirus (HPV), hepatitis B surface antigen (HBsAg), hepatitis C surface antigen (HCVsAg), or venereal disease research laboratory (VDRL). A skin biopsy was not possible because the patient was unwilling to cooperate, and the family declined owing to the sensitivity of the lesion site. Cutaneous examination revealed multiple flat-topped and round, shiny, erythematous, and moist papules ranging in size from 4 to 15 mm, along with a few verrucous nodules. Several lesions had coalesced to form plaques extending to the posterior part of the scrotum (Figure 1).

Based on the clinical findings, a diagnosis of PPN was established. During a four-month follow-up period, gradual spontaneous resolution of the skin lesions was observed. The patient and caregivers were educated on the importance of maintaining proper aeration of the affected area and minimizing irritation. Preventive measures, including regular diaper changes and the use of barrier creams such as zinc oxide, were emphasized. Additionally, topical tacrolimus 0.03% was prescribed to aid in the resolution of the lesions.

## Discussion

Goldberg et al. first described the term PPN in 1992 [2]. The frequency of irritant contact dermatitis among anogenital symptoms has been estimated at around one-fifth. Diaper dermatitis is an inflammatory reaction caused by direct contact with feces, urine, or both, which usually presents as an erythematous rash with micropapules and scaling. In skin persistently exposed to feces or urine, uncommon clinical patterns such as erosive dermatitis, granuloma gluteale infantum, or pseudoverrucous lesions have also been described [6]. It was observed that the lesions appeared between one week and 10 months after the start of dermatitis. Factors responsible for PPN include urinary incontinence, toilet paper, detergents, and insufficient diaper changes [7]. PPN may be associated with poor socioeconomic status and inadequate hygiene [8].

Several pathogens play a role in diaper dermatitis. Prolonged contact with feces and urine causes the skin to become overhydrated, which is a significant precipitating factor. Maceration induced by an ongoing localized wet environment, further exacerbated by impermeable diaper covers, makes the skin more permeable and vulnerable to primary irritant damage. Among the irritant fecal substances are proteases and lipases released by the pancreas, and bile salts enhance the action of lipases. The role of ammonia is indirect, although it has long been considered a primary irritant. With an elevated pH, fecal enzymes become more potent and harmful. Ureases produced by intestinal bacteria generate ammonia from urinary urea [7].

A differential diagnosis may include infectious warts, condyloma lata, condyloma acuminata, neoplastic processes, halogenoderma, bacterial infections, candidiasis, cutaneous Crohn's disease, and Langerhans cell histiocytosis [9]. It was described as wart-like papules or small, gray-white or reddish-brown erosive papules with a diameter of 2 mm to 3 mm. The histopathology reveals psoriasiform epidermal hyperplasia with acanthosis, broad hyperparakeratosis, papillary edema, prominent dermal blood vessels, and mild to moderate perivascular infiltrates [2]. PPN may be distinguished from others based on the history of sexual contact, detailed cutaneous findings, and certain investigations, such as rapid plasma reagin (RPR), VDRL, bacterial and fungal cultures, and skin biopsy [8].

Restoring the function of the skin barrier and eliminating the precipitating factors should be the main aim of treatment. Therefore, changing diapers regularly is essential. Disposable superabsorbent diapers are advised as preferred to cloth diapers since the former become wet quicker and can contain irritant detergents. It has been reported that PPN can be effectively treated with repeated cryotherapy treatments and shave excision. However, these surgical or damaging techniques only offered short-term comfort. Topical zinc oxide and steroids are used as protective barriers. A recent study evaluated the application of potato protease inhibitors as a novel treatment for the prevention of protease-induced perianal dermatitis [2].

It has been suggested by several authors that the terms pseudoverrucous papules, granuloma gluteale infantum, and Jacquet's erosive napkin dermatitis overlap and can be used alternatively. In order to encompass all of these variations, the term "erosive papulonodular dermatosis" was created [8].

## Conclusions

This case report presented PPN in a child with multiple congenital conditions and chronic incontinence, emphasizing the importance of early recognition and management. PPN can mimic dermatological and infectious conditions, leading to misdiagnosis and delayed treatment. Effective treatment focuses on eliminating moisture exposure, maintaining skin aeration, and using protective barriers. Key strategies include frequent diaper or ostomy changes, gentle cleansing, and barrier creams like zinc oxide. Topical therapies, including tacrolimus or mild corticosteroids, may aid resolution.
